# Investigation of the high rates of extrapulmonary tuberculosis in Ethiopia reveals no single driving factor and minimal evidence for zoonotic transmission of *Mycobacterium bovis* infection

**DOI:** 10.1186/s12879-015-0846-7

**Published:** 2015-03-03

**Authors:** Stefan Berg, Esther Schelling, Elena Hailu, Rebuma Firdessa, Balako Gumi, Girume Erenso, Endalamaw Gadisa, Araya Mengistu, Meseret Habtamu, Jemal Hussein, Teklu Kiros, Shiferaw Bekele, Wondale Mekonnen, Yohannes Derese, Jakob Zinsstag, Gobena Ameni, Sebastien Gagneux, Brian D Robertson, Rea Tschopp, Glyn Hewinson, Lawrence Yamuah, Stephen V Gordon, Abraham Aseffa

**Affiliations:** Animal and Plant Health Agency, TB Research Group, New Haw, Addlestone, Surrey, KT15 3NB UK; Swiss Tropical and Public Health Institute, PO Box CH-4002, Basel, Switzerland; Armauer Hansen Research Institute, PO Box 1005, Addis Ababa, Ethiopia; Aklilu Lemma Institute of Pathobiology, Addis Ababa University, PO Box 1176, Addis Ababa, Ethiopia; Center for Molecular Bacteriology and Infection, Department of Medicine, Flowers building, South Kensington, Imperial College London, London, SW7 2AZ UK; UCD Schools of Veterinary Medicine, Medicine and Medical Science, Biomolecular and Biomedical Science and UCD Conway Institute, University College Dublin, Dublin, Republic of Ireland; University of Basel, Basel, Switzerland; University of Würzburg, Institute for Molecular Infection Biology, Würzburg, Germany

**Keywords:** Mycobacterium, Tuberculosis, Bovis, Pulmonary, Extrapulmonary, Lymphadenitis, Zoonotic, Ethiopia

## Abstract

**Background:**

Ethiopia, a high tuberculosis (TB) burden country, reports one of the highest incidence rates of extra-pulmonary TB dominated by cervical lymphadenitis (TBLN). Infection with *Mycobacterium bovis* has previously been excluded as the main reason for the high rate of extrapulmonary TB in Ethiopia.

**Methods:**

Here we examined demographic and clinical characteristics of 953 pulmonary (PTB) and 1198 TBLN patients visiting 11 health facilities in distinct geographic areas of Ethiopia. Clinical characteristics were also correlated with genotypes of the causative agent, *Mycobacterium tuberculosis.*

**Results:**

No major patient or bacterial strain factor could be identified as being responsible for the high rate of TBLN, and there was no association with HIV infection. However, analysis of the demographic data of involved patients showed that having regular and direct contact with live animals was more associated with TBLN than with PTB, although no *M. bovis* was isolated from patients with TBLN. Among PTB patients, those infected with Lineage 4 reported “contact with other TB patient” more often than patients infected with Lineage 3 did (OR = 1.6, CI 95% 1.0-2.7; p = 0.064). High fever, in contrast to low and moderate fever, was significantly associated with Lineage 4 (OR = 2.3; p = 0.024). On the other hand, TBLN cases infected with Lineage 4 tended to get milder symptoms overall for the constitutional symptoms than those infected with Lineage 3.

**Conclusions:**

The study suggests a complex role for multiple interacting factors in the epidemiology of extrapulmonary TB in Ethiopia, including factors that can only be derived from population-based studies, which may prove to be significant for TB control in Ethiopia.

**Electronic supplementary material:**

The online version of this article (doi:10.1186/s12879-015-0846-7) contains supplementary material, which is available to authorized users.

## Background

There were approximately 8.6 million new cases and 1.3 million deaths due to tuberculosis (TB) in 2012 [1]. Although pulmonary TB is the most common manifestation, an estimated one million people (~15%) develop extrapulmonary TB, of which TB lymphadenitis in the cervical lymph nodes (TBLN) is the most frequent form [2,3]. Failure to diagnose and treat TBLN can lead to serious health consequences such as disseminated TB [2].

The majority of all human TB cases are caused by *Mycobacterium tuberculosis* but the closely-related *Mycobacterium bovis*, the causative agent of TB in cattle and a range of domestic and wild animals, can also cause disease in humans. Zoonotic transmission can occur through the aerosol route during close contact with animals leading to pulmonary disease [4], but *M. bovis* is primarily transmitted through consumption of contaminated milk and is often associated with TBLN [5].

**Figure 1:**
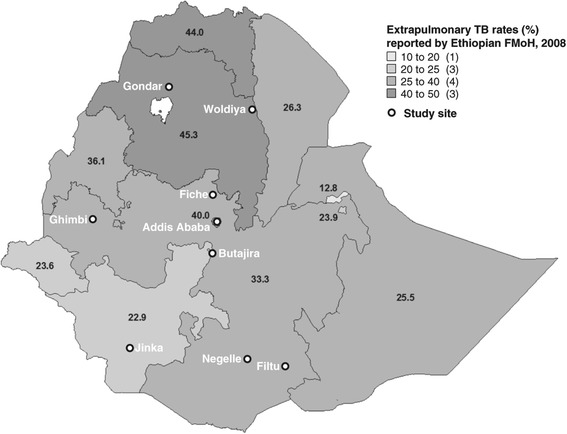
**Mapping of collection sites and extrapulmonary TB in Ethiopia.** Information received from the Federal Ministry of Health (FMoH), Ethiopia.

Ethiopia, with a population of over 90 million people, is among the countries with the highest TB burdens in the world, with an incidence rate of 247 per 100,000 in 2012 [1]. Moreover, Ethiopia has reported a higher than average incidence of extrapulmonary TB since records started in the 1990s. What are the risk factors that can explain this high rate of extrapulmonary TB in Ethiopia? More than 80% of all extrapulmonary cases involve cervical TB lymphadenitis that currently accounts for around 33% of all incident TB cases in the country, with a prevalence that roughly increases from 20% to 45% along a south to north geographic axis (data from Ethiopian Federal Ministry of Health; Figure 1). In parallel, Ethiopia is home to the largest livestock population in Africa with ~52 million cattle [6], and more than 80% of the labour force works in the agricultural sector. As several investigations have shown that bovine TB is endemic in Ethiopian cattle [7,8] and reaches high prevalence in regions with intensive husbandry systems [9-11], it would seem plausible that zoonotic transmission of *M. bovis* would have an influence on the prevalence of TBLN in the country. However, in a large nation-wide molecular epidemiology study [12] we explored the public health risk for zoonotic TB in Ethiopia and concluded that the overall role of *M. bovis* as a causative agent of TB in humans was approximately only 1%. This led us to conclude that the high incidence rate of extrapulmonary TB reported in Ethiopia is likely due to other factors.

In our molecular epidemiological study [12], nearly 1,000 mycobacterial strains were isolated from over 2,000 patients nationwide (Figure 1) who were diagnosed with either TB lymphadenitis or pulmonary TB. In parallel to identifying the pathogen population structure, demographic and clinical data were collected from the same patients. Here we report the results of our analyses of these demographic and clinical data in an attempt to elucidate the factors that influence rates of extrapulmonary TB in Ethiopia and to inform public health control measures generally.

## Methods

### Selection of study sites

The location of the study sites is shown in Figure 1 with Gondar, Woldiya, Ghimbi, Fiche, and Butajira in the Ethiopian highlands where subsistence farming dominates, while Negelle, Filtu, and Jinka (“NFJ”; referred to as one site in this paper) are located in the southern parts of the country where people are mainly pastoralists or agro-pastoralists. Fine needle aspirates (FNA) and sputum samples, for diagnosis of TBLN and pulmonary TB respectively, were collected from patients attending hospitals in Gondar, Woldiya, Ghimbi, Butajira and Negelle. In addition, sputum samples were collected in hospitals at Fiche, Jinka and Filtu, as well as health centres at three suburban sites of Addis Ababa where residents are mainly engaged in subsistence farming or in intensified dairy farming (Holeta, Sululta, and Chancho).

### Collection of specimens and demographic and clinical information

All consecutive and consenting patients with pulmonary TB or TBLN presenting at hospitals or health centres located in the study sites were recruited. Patients with disseminated TB (e.g. evidence of combined lung and lymph node disease) were excluded from the study. Demographic and clinical information (including cardinal symptoms and their duration: fever, night sweat, weight loss, poor appetite, weakness, cough, and blood in sputum as well as information on nodes such as number, size, and consistency) was collected from each patient through an interview with a clinician using the same questionnaire in all health centres. Sputum samples were collected from newly diagnosed pulmonary TB patients confirmed by the health facility laboratory to have acid-fast bacilli (AFB) in the sputum. The Ethiopian national algorithm (2008; Additional file 1: Figure S1) was used for recruitment of TB lymphadenitis patients, and patients diagnosed with TBLN had an opportunity to be serologically tested for HIV (no information on HIV status for pulmonary TB cases was recorded in this study). FNA material was collected aseptically from enlarged cervical lymph nodes with a 21-gauge needle attached to a 10 ml syringe and smeared onto glass slides for routine cytology. The remaining material in the syringe was rinsed into a tube with 1 ml PBS solution and used for culture. Sputum and FNA samples were stored at 4°C at the field sites and during transportation to the TB laboratory of the Armauer Hansen Research Institute (AHRI) in Addis Ababa where they were further processed within five days after sampling. For remote collection sites (NFJ), samples were commonly stored at the field site at −20°C until transport to AHRI. Informed consent was obtained from all patients enrolled in the study. All participating health facilities and regional health bureaus provided support letters and ethical approval was obtained from the AHRI and the All Africa Leprosy, Tuberculosis and Rehabilitation Training Centre (ALERT) Ethics Review Committee, regional health bureau ethics committees and the Ethiopian National Research Ethical Review Committee.

### Cytology and HIV testing

FNA smears were air dried on glass slides (at the collection site) and brought to AHRI for Ziehl-Neelsen and Wright-staining (BDH chemicals Ltd, England). Cytological examination was performed by an experienced pathologist for evidence of pathology indicative of tuberculosis and the results reported to the respective health facilities for appropriate patient management. HIV testing was done according to national guidelines at the health facility where the patient was treated for TB and test procedures followed manufacturer’s instructions. Up to three different tests were used: KHB rapid HIV; Stat-Pak HIV; and Uni-gold HIV [13].

### Culture and molecular typing

The procedures used for isolation and typing of the causative agents from the recruited patients as well as the molecular epidemiology outcomes have been published elsewhere [7,12]. In brief, FNA and sputum samples collected from patients in this study were processed and cultured at 37°C on three different media, including two Löwenstein-Jensen (LJ) media (supplemented with either glycerol or pyruvate) and a modified Middlebrook 7H11 medium optimised for the culture of *M. bovis* [12,14]. Slants with no growth at week 8 were considered negative. Bacterial colonies from culture-positive samples were Ziehl-Neelsen stained according to a standardized protocol to identify AFB. Cultures positive for AFB were prepared as 20% glycerol stocks and stored at −80°C. In parallel, heat-inactivated AFB positive samples were investigated by multiplex PCR for Large Sequence Polymorphism (LSP; *e.g.* RD4 and RD9) regions and by lineage-specific single-nucleotide polymorphism (SNP) analysis [7,15]. Isolates genetically typed as belonging to the *M. tuberculosis* complex were spoligotyped as previously described [16].

### Statistical analysis

All data collected from patients and the laboratory were double-entered into a Microsoft Access database and the entry errors corrected with EpiInfo 3.5.1 (data compare utility). Questionnaire and laboratory data were linked by a unique identification code. Analyses of data were done in STATA/IC 10 for Windows (StataCorp, College Station, TX). Logistic regression models were used for univariate analyses of crude odds ratios (OR) of demographic and clinical data and models were also adjusted to age group (≤20 years, >20 to ≤45 years, >45 years), sex and site (seven sites) to generate ORs adjusted to expected confounders ORs of demographic and potential risk factors were further compared to those obtained in the full model with all variables. We created binary outcome variables for Lineages 3, 4 and 7 (the respective lineage vs. all other lineages) to test associations with explanatory variables such as pulmonary TB and TBLN. The statistical significance threshold was set at 0.05, p ≤ 0.05.

## Results

### Study population and Sample collected

During a period of five years from 2006–2010, we screened 2151 patients with untreated clinically diagnosed pulmonary TB and TB lymphadenitis (TBLN) in seven sites distributed across Ethiopia (Figure 1). Patients attending clinics in Gondar and Woldiya were predominantly Amhara, while the majority of people in Ghimbi, Fiche and the suburban clinics of Addis Ababa were Oromo. The population in Butajira was mainly Gurage, and clinics in the south (Negelle/Filtu/Jinka) included people of Oromo, Somali, Amhara, and Ari ethnic groups.

Clinics at Fiche and Addis Ababa did not use fine needle aspirates (FNA) for diagnosis of TBLN and therefore only sputum from pulmonary TB cases was collected from these sites. The yield of AFB positive cultures differed between sites, likely reflecting logistic issues associated with sample transport (Table 1).

**Table 1:** **Patient numbers enrolled and AFB culture-positivity per collection site**

**Collection site**	**Pulmonary TB**		**TB lymphadenitis**	
	**Patients**	**Culture-positive**	**Patients**	**Culture-positive**
Gondar	120	103 (86%)	156	46 (29%)
Woldiya	36	30 (83%)	333	160 (48%)
Ghimbi	68	65 (96%)	264	99 (38%)
Fiche	223	203 (91%)	0	-
Addis Ababa	122	76 (62%)	0	-
Butajira	101	99 (98%)	360	138 (38%)
Negelle/Filtu/Jinka	283	180 (64%)	85	13 (15%)
Total	953	756 (79%)	1198	456 (38%)

### Bacterial culture and clinical features

Acid-fast bacilli were isolated from 1212 of the 2151 patients (Table 1). As expected, AFB positive cultures were obtained more frequently from the 953 patients with smear-positive pulmonary disease than from the 1198 patients with suspected TBLN, with an average yield between the collection sites of 79% and 38% culture positivity, respectively. In comparison, Ziehl-Neelsen staining of 302 FNA samples demonstrated only 10% smear-positivity, reflecting challenges in diagnosis of TBLN by different methods. However, the frequency of positive TBLN cultures was higher in FNA samples that showed cytological evidence of tuberculosis (220 positive cultures from 469 samples; 47%) than those with negative cytology (23 positive cultures from 131 samples; 18%).

Our analysis of clinical features of patients diagnosed with TBLN and pulmonary TB with regard to culture outcome can be found in Additional file 2: Table S2 and Additional file 3: Table S3A. Constitutional symptoms (*i.e.* fever, night sweat, weight loss, poor appetite, and weakness) were more frequent in patients where a positive culture could be confirmed, reaching statistical significance for “night sweat” and “weakness” for patients of both disease outcomes compared to those negative on culture. For patients diagnosed with TBLN, significant differences were also noticed in culture positivity related to perceived increased rate and duration of neck swelling; culture positivity was higher in “fast” swelling as compared to “slow” swelling of the cervical lymph nodes (38% versus 54%) (Additional file 3: Table S3), and with regard to the duration, TBLN patients seeking health care within one month of the appearance of swelling were significantly more likely to be culture-positive than those who were examined only one year after appearance. Culture-positivity also tended to increase in proportion to node size; 103/288 (36%) of samples from lymph nodes less than 1 cm yielded positive cultures, in comparison to 58/137 (42%) of samples from lymph nodes greater than 4 cm [adjusted OR (95% CI) 2.3 (0.9-5.8)]. The median duration of swelling recorded for all recruited TBLN patients was 12 weeks and the average size of swollen lymph nodes was approximately 3 cm. For additional results on clinical features, see Additional file 2: Table S2 and Additional file 3: Table S3.

Patients diagnosed with TBLN at the clinics had an opportunity to be serologically tested for HIV. We obtained 395 records for HIV status, of which 14 (3.6%) were HIV positive. Five of these 14 HIV positive patients were confirmed with TBLN by positive mycobacterial culture. No HIV data were collected from pulmonary TB patients.

### Demographic information

In an attempt to elucidate risk factors associated with disease type, we then compared 626 patients with pulmonary TB and 328 with TBLN for their demographic characteristics (Table 2). Having regular and direct contact with live animals, was a significant risk factor for TBLN when compared to pulmonary TB. In addition, an association was observed between education and TBLN, with patients having a degree from secondary school or higher education being at lower risk of developing TBLN in comparison to pulmonary TB (Table 2). We also analysed our data within each study site to investigate if there was any association between ethnic group and disease outcome. This could not be analysed on a national level as sites and ethnic groups were highly correlated and affected by sampling bias. No associations between ethnic groups and TBLN or pulmonary TB were found (data not shown).

**Table 2:** **Demographic characteristic of 954 culture-positive patients with identified strain**

		**PTB**		**TBLN**		**Crude OR**	**Adjusted OR**
		**n**	**%**	**n**	**%**	**95% CI**	**95% CI**
**Age** ^a^	≤20 years	128	51.6	120	48.4	1	1
	>20 - ≤45 years	355	70.6	148	29.4	0.4 (0.3-06.)***	0.5 (0.4-0.8)**
	>45 years	87	80.6	21	19.4	0.3 (0.2-0.4)***	0.3 (0.2-0.6)***
**Sex** ^b^	Male	331	70.6	138	29.4		
	Female	246	59.6	167	40.4	1.6 (1.2-2.2)***	1.6 (1.1-2.3)*
**Education**	Illiterate	311	67.2	152	32.8	1	1
	Primary	158	58.5	112	41.5	1.5 (1.1-2.0)*	0.7 (0.4-1.1)
	Secondary	86	71.7	34	28.3	0.8 (0.5-1.3)	0.3 (0.2-0.6)***
	Higher degree	22	81.5	5	18.5	0.5 (0.2-1.3)	0.2 (0.1-0.7)**
**Previous TB contact**	No	331	59.8	223	40.2		
	Yes	154	66.7	77	33.3	0.7 (0.5-1.0)	0.8 (0.5-1.3)
**Intake of raw milk**	No	221	66.4	112	33.6		
	Yes	353	64.6	193	35.4	1.1 (0.8-1.4)	1.9 (1.2-2.8)**
**Living with animals in same household**	No	289	70.2	123	29.8		
	Yes	273	60.7	177	39.3	1.5 (1.1-2.0)**	1.7 (1.1-2.6)*
**Regular and direct contact with live animals**	No	214	72.0	83	28.0		
	Yes	327	64.9	177	35.1	1.4 (1.0-1.9)*	3.0 (1.9-4.5)***
**BCG vaccination**	No	329	82.5	70	17.5		
	Yes	89	77.4	26	22.6	1.4 (0.8-2.3)	1.6 (0.8-3.3)

^a^adjusted for sex and site alone; ^b^adjusted for age and site alone; *p ≤ 0.05; **p ≤ 0.01; ***p ≤ 0.001.

### The role of *M. tuberculosis* lineages in clinical presentation

**Figure 2:**
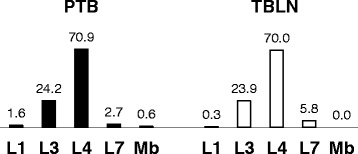
**Representative lineages (in percentage) of** ***M. tuberculosis complex*** **strains collected from TB lymphadenitis and pulmonary TB patients in Ethiopia (data taken from Firdessa** ***et al.*** **[** 10 **]). Mb,** ***M. bovis)*** **PTB = pulmonary TB, TBLN = tuberculous lymphadenitis.**

The *M. tuberculosis* complex strains that caused TB in the 954 patients have been previously reported by Firdessa et al. [12]. A summary of the lineage distribution of these strains is shown in Figure 2, stratified by TBLN and pulmonary TB cases, respectively. Here we have made use of this information to seek correlations between demographic/clinical features and lineages of *M. tuberculosis*. Demographic and clinical data from the 328 patients with TBLN and the 626 cases with pulmonary TB were therefore stratified by *M. tuberculosis* lineage. Due to low statistical power for lineages with limited isolates, only Lineage 3 and Lineage 4 were compared individually to all other lineages (Additional file 2: Table S2A and Additional file 3: Table S3A). Analysis of demographic factors among pulmonary TB patients showed a borderline statistical difference between TB contact and *M. tuberculosis* lineage causing the disease; patients infected with Lineage 4 reported “contact with other TB patient” more often than patients infected with Lineage 3 did (OR = 1.6, CI 95% 1.0-2.7; p = 0.064). The analogous comparison in TBLN patients showed no significant difference. Moreover, pulmonary TB patients infected with Lineage 4 strains – in contrast to Lineage 3 – tended to have a more severe manifestation (OR > 1) for a majority of the constitutional symptoms (Additional file 2: Table S2A and Additional file 3: Table S3A). Fever was the most distinct symptom in this regard and it was further enhanced when data on type of fever was included (data not shown); high fever, in contrast to low and moderate fever, was significantly associated with Lineage 4 (OR = 2.3; p = 0.024). Corresponding data collected from the TBLN patients generated a similar but reverse result: TBLN cases infected with Lineage 4 tended to get milder symptoms overall for the constitutional symptoms than those infected with Lineage 3.

## Discussion

This study identified no major patient or bacterial strain factor that alone could be responsible for the high rate of TBLN in Ethiopia. In addition, no association was found with HIV infection.

The incidence of extrapulmonary TB, of which the vast majority is TBLN, among TB patients in Ethiopia has steadily increased since the 1990s, reaching an average of around 33% across the country. Iwnetu et al. [17] questioned if this increase was simply due to over-diagnosis, with their study concluding that up to 15% of all TBLN cases could be wrongly diagnosed; however, this clearly does not completely explain the high incidence of extrapulmonary TB. We recently tested the hypothesis that TBLN in Ethiopia would be associated with zoonotic transmission of *M. bovis* from cattle [12]. Molecular identification of the causative agents showed that the contribution of *M. bovis* to human TB was less than 1%. Even more unexpected was the fact that no TB lymphadenitis patient was diagnosed with *M. bovis* infection, but instead, the four human cases identified with *M. bovis* infection all had pulmonary TB. This suggests that other risk factors need to be considered to explain the high incidence of extrapulmonary TB in Ethiopia [18,19]. To search for additional risk factors, our extensive health centre-based survey was also designed to overlap geographically with areas previously examined for bovine TB in cattle [7,10,20,21] and to include sites distributed across the documented south–north gradient (20-45%) of extrapulmonary TB (Figure 1). Based on further analysis of our epidemiological study presented here and elsewhere [7,12], we suggest a set of possible factors that may influence the high incidence of extrapulmonary TB as well as the lower than expected burden of *M. bovis* infections in humans.

### Zoonotic TB – *M. bovis*

The low incidence of human infection by *M. bovis* may simply reflect a relatively low prevalence of bovine TB. In the 1930s and 40s, it was estimated that 30-40% of cattle in England and Wales had TB, with zoonotic transmission suggested to account for around 5-6% of the human TB burden [22,23]. In comparison, prevalence rates of 1-10% for bovine TB in Ethiopian zebu cattle grazing in pasture are relatively low [7,21] and may therefore have a lower impact on the overall contribution to human TB in Ethiopia.

The low rate of isolation of *M. bovis* in humans recorded by us [12] can be compared to other reports that used genotyping of isolates for definitive characterisation. Recent studies identified 3.5% of human TB in Madagascar as caused by *M. bovis* [24], but found no *M. bovis* in Côte d’Ivoire [25], in Brazil [26], or in Chad [27] despite the fact that these countries are endemic for bovine TB in cattle [28-32]. Higher rates were found in Mexico, with separate studies identifying 6% and 28% of human mycobacterial isolates as *M. bovis* [33,34]. Taken together, these findings suggest a low overall rate of zoonotic transmission of *M. bovis*, particularly in Africa, but do not exclude this as a public health concern in selected areas. Of particular note in the context of the present study is that we did not sample TBLN cases in urban areas where there are high rates of bovine TB associated with intensified dairy farming [9,11,35]. At a global level, systematic surveys in additional geographic regions will be important in making a realistic assessment of the human health risk posed by zoonotic transmission of *M. bovis*. The high frequency of *M. bovis* isolated from Mexican patients may be linked to the low overall TB incidence rate (17/100,000), and it can be anticipated that the relative contribution of zoonotic transmission will increase as control programmes for humans progress towards TB elimination, particularly when programmes against bovine TB in cattle are lagging behind [36].

Accurate speciation of the causative agent in individuals with TB is not trivial and demands time and resources. Although newly developed PCR-based techniques can identify specific mycobacteria directly from infected human specimens [37], isolation of the bacteria by culturing - prior to further characterisation – is considered as the diagnostic gold standard due to significantly higher sensitivity and specificity. We do not think that our low rates of *M. bovis* isolation from humans were simply due to sample processing techniques that selected against *M. bovis*; we routinely cultured *M. bovis* from cattle samples from the same sites using the same protocols. The traditional method to distinguish between species within the *M. tuberculosis* complex has primarily been biochemical testing based on phenotypic differences such as nitrate reduction [38] and pyrazinamide resistance [39]. However, the reliability of phenotype-based assays to accurately distinguish between *M. tuberculosis* and *M. bovis* has been contested [40,41]. Therefore, reservations regarding conclusions of earlier studies based solely on biochemical tests or cultural characteristics are justified. The currently well-established genotyping methods (*e.g*. LSP and SNP typing), as used in this study, should be a requirement for definitive differentiation of species from the *M. tuberculosis* complex.

### Zoonotic TB – *M. tuberculosis*

From our initial hypothesis that the high prevalence of extrapulmonary TB may reflect zoonotic transmission, we anticipated that TBLN patients might have close contact with farm animals and/or consume raw milk. This was in fact the case. Our demographic analysis identified contact with livestock as a risk factor for TBLN when compared to pulmonary TB. Given the large cattle population in Ethiopia (~50 million) and that humans and cattle live in close proximity, together with reports that *M. tuberculosis* has been isolated both from cattle [7,42,43] and milk samples [44], there would appear to be ample opportunity for direct involvement of livestock in the transmission cycle of *M. tuberculosis*. In our study of bovine TB in Ethiopia based on visual inspection of carcasses and culture of suspect lesions, we obtained 8 *M. tuberculosis* isolates from 32,800 animals [7], corresponding to a “culture-proven” prevalence of 24 cases per 100,000. In comparison, the prevalence of smear-positive TB in humans is 74 cases per 100,000 in Ethiopia [1]. In addition, the comparative intradermal skin test that is used to assess bovine TB prevalence may overestimate true prevalence of *M. bovis* infection as the test cannot distinguish between cattle infected with *M. bovis* or *M. tuberculosis*. In fact, a recent Ethiopian study isolated both *M. bovis* and *M. tuberculosis* at similar rates from skin-test reactor cattle in small-holder farms owned by households with a TB index case [43]. Taken together, even if the extent of cattle infected with *M. tuberculosis* is unknown it should be considered as a potential risk factor for human transmission, with for example ingestion of contaminated milk being one possible source of infection. The contribution of zoonotic transmission of *M. tuberculosis* to the high rate of extrapulmonary TB remains to be clarified. As epidemiological data from Ethiopia suggest a higher prevalence of *M. bovis* in cattle as compared to *M. tuberculosis*, one would expect that the rate of zoonotic transmission of *M. bovis* would be higher than that for *M. tuberculosis*. However, several unanswered questions remain to be solved in this human-cattle-human transmission cycle, including transmission routes and rates as well as differences in pathogenicity between *M. bovis* and *M. tuberculosis* in cattle and humans.

### Lineage and disease presentation

Having ruled out *M. bovis* as a cause for the high incidence of TBLN, we can ask whether particular features of the circulating *M. tuberculosis* strains might play a role. An association of strains belonging to *M. tuberculosis* Lineage 4 with pulmonary TB as opposed to extrapulmonary disease in the form of TB meningitis has been described in Vietnam [45]. Similarly, Lari et al. [46] have shown an association between Lineage 3 and extrapulmonary TB. Across Ethiopia as a whole, we did not observe any association between *M. tuberculosis* lineage and disease presentation (Figure 2). Increased representation of Lineage 4 in contrast to Lineage 3 in TBLN (data not shown) was however observed in the north of the country but it only reached statistical significance in Gondar (Table 3). Lineage differences were also identified by comparison of disease severity indicators; infection with Lineage 4 was weakly associated with increased severity in pulmonary TB patients, and with decreased severity in TBLN. While these results are consistent with the hypothesis that genetic diversity of *M. tuberculosis* is associated with phenotypic diversity linked to disease presentation [47-49], these differences do not provide an obvious explanation for the high overall rate, or for the North–south gradient, of extrapulmonary TB in Ethiopia.

**Table 3:** **Representation of** ***M. tuberculosis*** **Lineage 4 among TB lymphadenitis and pulmonary TB cases from respective collection sites**

	**PTB**		**TBLN**		**Crude OR**	
	**N**	**%**	**n**	**%**		
**Gondar**	60	84.5	11	15.5		
	32	60.4	21	39.6	3.6 (1.5-8.3)**	0.003
**Woldiya**	14	20.0	56	80.0		
	9	14.3	54	85.7	1.5 (0.6-3.8)	0.4
**Ghimbi**	7	38.9	11	61.1		
	40	40.8	58	59.2	0.9 (0.3-2.6)	0.9
**Butajira**	10	34.5	19	65.5		
	60	39.7	91	60.3	0.8 (0.3-1.8)	0.6
**Negelle/Filtu/Jinka**	38	95.0	2	5.0		
	123	96.1	5	3.9	0.8 (0.1-4.1)	0.8

**p ≤ 0.01 PTB = pulmonary tuberculosis; TBLN = tuberculous lymphadenitis; OR = odds ratio.

Adjusted OR; adjusted for site (n = 7), age category (3) and sex (binary). L3 = Lineage 3 (CAS); L4 = Lineage 4 (Euro-American); L7 = Lineage 7 (new).

There is evidence of distinct geographic structuring of *M. tuberculosis* populations in Ethiopia. Representation of Lineage 3 was up to four times higher in the north as compared to the other sites. A recent study in neighbouring Sudan also reported a high frequency of Lineage 3, with SIT 25 (the predominant Lineage 3 spoligotype in Ethiopia) accounting for almost half of the *M. tuberculosis* isolates [50]. In contrast, Lineage 1, which has been reported at high frequency in Somalia (~50% (129/256); SITVIT2), was isolated only from sites in southern Ethiopia. In comparison to Ethiopia, Sudan (25%) and Somalia (20%) reported lower case notifications of extrapulmonary TB in 2012 [1]. Based on these reports from Sudan and current data from Ethiopia, we cannot propose a correlation between a high rate of extrapulmonary TB and infection with Lineage 3 (subtype SIT 25) strains; this is also shown by our observation that Lineage 4 (rather than Lineage 3) is associated with TBLN in Gondar. Similarly, the two major subtypes of Lineage 4 (Spoligotypes SIT 149 and SIT 53) were relatively well distributed among the collection sites in Ethiopia and no strong clustering towards the north (*e.g.* Gondar) was observed. Thus, no obvious relationship between subtype and disease manifestation was found, but such a correlation cannot be excluded. We conclude that while *M. tuberculosis* genotype may have some effect on disease presentation, it cannot account for the overall elevation in the prevalence of extrapulmonary TB in Ethiopia.

### Other possible factors driving disease presentation

Co-infection with HIV is strongly associated with increased rates of extrapulmonary TB [51-53]. While the rate of HIV-TB co-infected patients in Ethiopia (10.2% of all incident cases) is higher than in neighbouring Sudan and Somalia (7.5% and 3.6% respectively), it is much lower than that in Kenya (39%), which has an incident rate of extrapulmonary TB (18%) approximately half of that reported for Ethiopia [1]. Working within national control programme guidelines in our present study, we obtained HIV infection data for 399 patients with TBLN; the rate of HIV-positivity (3.6%) was below the national average reported for all TB cases. Though limited number of patients were compared, we conclude that HIV co-infection does not explain the high rate of extrapulmonary TB, confirming observations of several previous studies in Ethiopia [17,54].

Data from the Ethiopian FMoH (2008) and analysed by us (data not shown) suggests that the total incidence of both TB and TBLN are positively associated with the proportion of urban population in Ethiopian regions. This likely reflects an urban population with easier access to health services and TB diagnostics than people living in more rural regions, and thus, may also influence the proportion of TBLN cases among all TB cases. Specifically, seeking health care may be particularly low for people with TBLN among rural populations with long distances from a health centre, as has been suggested for pastoralists living in the Afar region [55,56].

The Ethiopian human population is characterised by a high level of genetic diversity [57,58] and, although not studied here, host genetics may well have an influence on disease presentation [59]. In general, the incidence of extrapulmonary TB in North African countries is higher than that in Sub-Saharan Africa [1]. Assuming a human genetic effect, the high rates in Ethiopia may reflect the known admixture of northern and southern African human ancestry, and/or perhaps particular host and pathogen genotype combinations. Future analyses of disease outcomes as a function of different host-pathogen combinations may provide insights into the pathogenesis of TB and complement conventional genome-wide association studies [60,61].

A limitation of this study is that it is based on clinical cases visiting health facilities that enrolled smear positive pulmonary and cervical lymphadenitis TB cases. There was no prospective follow up to determine clinical outcome. Smear negative pulmonary and non-cervical extrapulmonary TB cases have not been included. Pulmonary TB cases were not tested for HIV. An epidemiological investigation with active case detection would have avoided the possible bias due to differential health seeking-behaviour and access to care. Nevertheless, our study provides very valuable insight into the epidemiology of TB in Ethiopia and will inform control in this and other high burden TB countries.

## Conclusions

Why Ethiopia stands out as a country with a high burden of extrapulmonary TB is enigmatic. Zoonotic transmission of *M. bovis* infection has been excluded as a major factor in TBLN, as was infection with HIV. Instead, the answer is likely to be more complex, and here we have presented several factors that could play significant roles, including zoonotic transmission of *M. tuberculosis* and genetic features of the pathogen and/or the host population. The study suggests a complex role for multiple interacting factors in the epidemiology of extrapulmonary TB in Ethiopia, including factors that can only be derived from population-based studies, which may prove to be significant for TB control in Ethiopia. We conclude that elucidation of the epidemiology of TBLN will contribute to a better understanding of the factors that maintain TB as a whole, rather than just pulmonary TB alone, and hence to improved disease control.

## Additional files

Additional file 1: Figure S1. Algorithms for recruitment of extrapulmonary TB (A) and pulmonary TB (B) patients according to the Ethiopian national TB and Leprosy control programme. Additional file 2: Table S2. Factors and clinical features of Pulmonary TB with regard to culture outcome (A) and *M. tuberculosis* lineage (B). Additional file 3: Table S3. Factors and clinical features of TB lymphadenitis with regard to culture outcome (A) and *M. tuberculosis* lineage (B).

## Abbreviations

TB: Tuberculosis
TBLN: TB lymphadenitis in the cervical lymph node
FNA: Fine needle aspirate
AFB: Acid fast bacilli
