# Supplementary material for: Investigation of the high rates of extrapulmonary tuberculosis in Ethiopia reveals no single driving factor and minimal evidence for zoonotic transmission of Mycobacterium bovis infection
Source: BMC Infect Dis. 2015 Mar 3;15:112. doi: 10.1186/s12879-015-0846-7 (PMC4359574; doi:10.1186/s12879-015-0846-7)
Supplement: Additional file 1: Figure S1. — Algorithms for recruitment of extrapulmonary TB (A) and pulmonary TB (B) patients according to the Ethiopian national TB and Leprosy control programme. [file 12879_2015_846_MOESM1_ESM.ppt]

## Slide 1
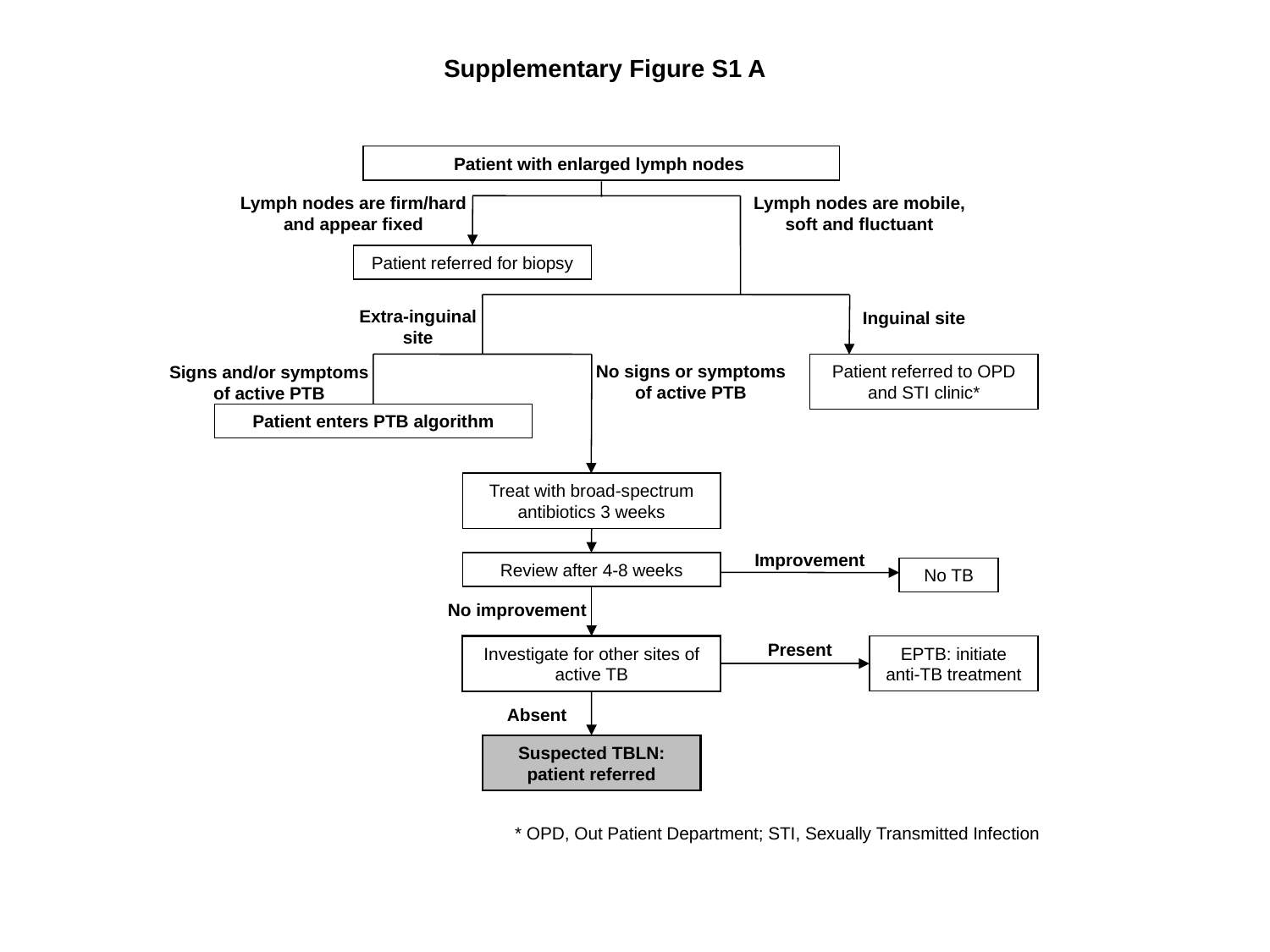

Supplementary Figure S1 A
Patient with enlarged lymph nodes
Lymph nodes are firm/hard and appear fixed
Lymph nodes are mobile, soft and fluctuant
Patient referred for biopsy
Extra-inguinal site
Inguinal site
No signs or symptoms of active PTB
Patient referred to OPD and STI clinic*
Signs and/or symptoms of active PTB
Patient enters PTB algorithm
Treat with broad-spectrum antibiotics 3 weeks
Improvement
Review after 4-8 weeks
No TB
No improvement
Present
Investigate for other sites of active TB
EPTB: initiate anti-TB treatment
Absent
Suspected TBLN: patient referred
* OPD, Out Patient Department; STI, Sexually Transmitted Infection

## Slide 2
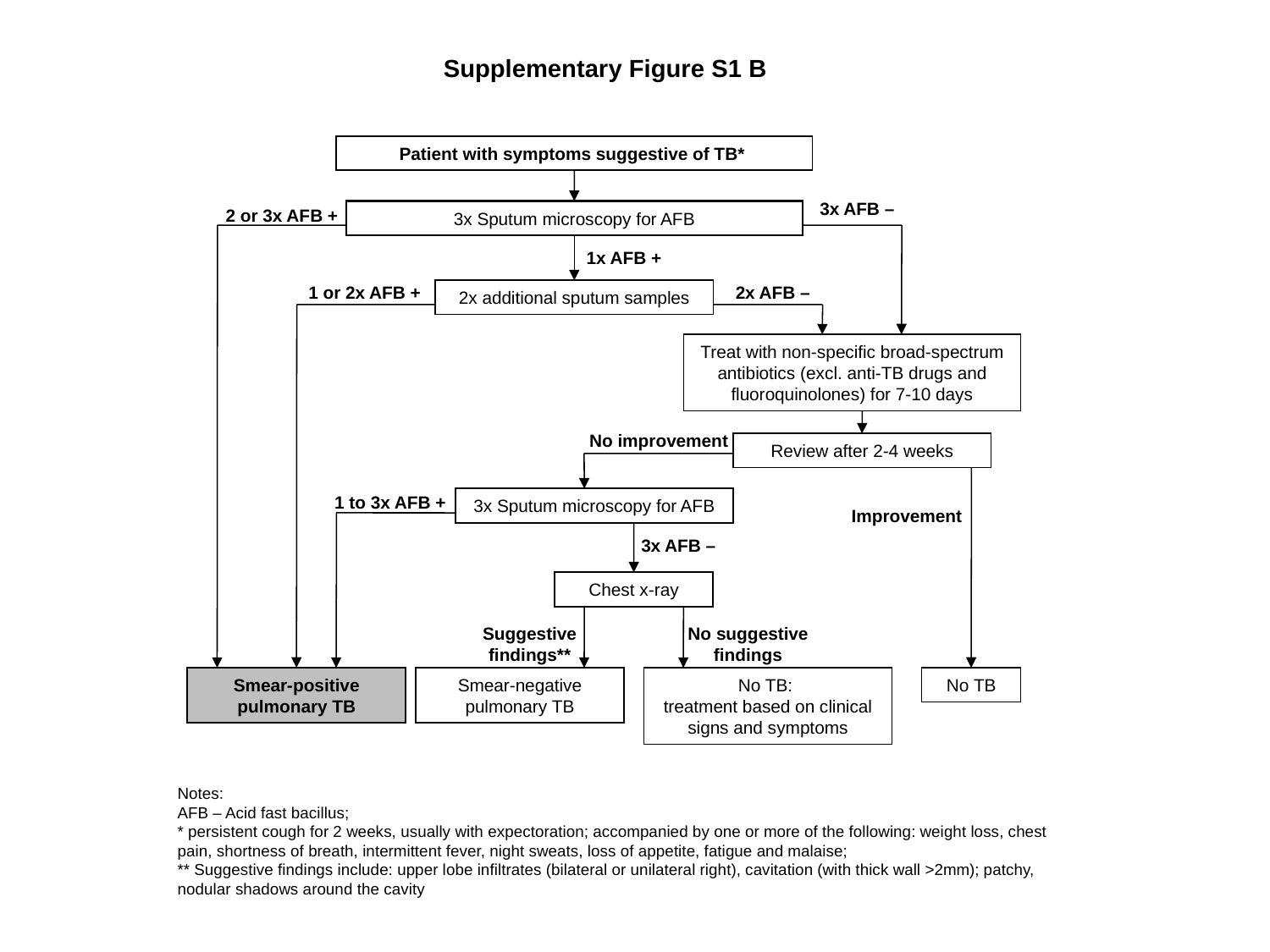

Supplementary Figure S1 B
Patient with symptoms suggestive of TB*
3x AFB –
2 or 3x AFB +
3x Sputum microscopy for AFB
1x AFB +
1 or 2x AFB +
2x AFB –
2x additional sputum samples
Treat with non-specific broad-spectrum antibiotics (excl. anti-TB drugs and fluoroquinolones) for 7-10 days
No improvement
Review after 2-4 weeks
1 to 3x AFB +
3x Sputum microscopy for AFB
Improvement
3x AFB –
Chest x-ray
Suggestive findings**
No suggestive findings
Smear-positive pulmonary TB
Smear-negative pulmonary TB
No TB:
treatment based on clinical signs and symptoms
No TB
Notes:
AFB – Acid fast bacillus;
* persistent cough for 2 weeks, usually with expectoration; accompanied by one or more of the following: weight loss, chest pain, shortness of breath, intermittent fever, night sweats, loss of appetite, fatigue and malaise;
** Suggestive findings include: upper lobe infiltrates (bilateral or unilateral right), cavitation (with thick wall >2mm); patchy, nodular shadows around the cavity
